# Efficacy and Safety of Iguratimod Supplement to the Standard Immunosuppressive Regimen in Highly Mismatched Renal Transplant Recipients: A Pilot Study

**DOI:** 10.3389/fimmu.2021.738392

**Published:** 2021-11-23

**Authors:** Jun Tao, Li Sun, Zijie Wang, Hao Chen, Zhijian Han, Hengcheng Zhang, Haiwei Yang, Zhengkai Huang, Shuang Fei, Xiaobin Ju, Ruoyun Tan, Min Gu

**Affiliations:** ^1^ Department of Urology, First Affiliated Hospital of Nanjing Medical University, Nanjing, China; ^2^ Transplantation Research Center, Renal Division, Brigham and Women’s Hospital, Harvard Medical School, Boston, MA, United States

**Keywords:** Iguratimod, randomized clinical trial, biopsy-proven acute rejection, donor-specific antibody (DSA), kidney transplantation

## Abstract

Iguratimod (IGU) can mitigate the symptoms of rheumatoid arthritis through its anti-inflammatory effects. The objective of this study was to investigate the clinical efficacy and safety of IGU in highly HLA-mismatched renal transplant recipients, in combination with standard immunosuppressive regimen. This pilot study was designed as an open-label, blank-control, randomized clinical trial on patients recruited from a single transplant center in China. Patients who met the inclusion criteria were randomized to the IGU (n=27) and blank control (n=27) groups. IGU was administrated with the conventional triple immunosuppressive protocol for 52 weeks after kidney transplantation. The incidence of biopsy-proven acute rejection rate was 14.8% (4/27) in the IGU group and 29.6% (8/27) in the control group, *P* = 0.19. The clinical rejection rate was also substantially reduced in the IGU group (3.7% *vs.* 18.5%, *P* = 0.08). *De novo* donor-specific antibody also showed a decline trend in the IGU group after 52 weeks. The graft function and incidence of adverse events were similar between the two groups. In addition, IGU intervention significantly decreased the number of NK cells throughout the follow-up. In conclusion, our study has shown the possibility that IGU could reduce the allograft rejection rate and *de novo* DSA with appreciable safety in combination with conventional immunosuppressants. Formal clinical trials were warranted based on current findings.

## Introduction

Kidney transplantation is the optimal treatment for end-stage renal failure and improves survival and quality of life in most cases (1). Although T cell-targeting immunosuppressive drugs have significantly reduced the occurrence of T cell-mediated rejection (TCMR) and prolonged graft survival among the recipients, they increase the risk of opportunistic infections and tumorigenesis. The role of humoral immunity in organ transplantation and graft rejection is less known. *De novo* donor-specific antibody (DSA) and non-human lymphocyte antigen (HLA) antibodies are primary mediators of antibody-mediated rejection (ABMR) and early graft dysfunction (2). Preformed DSA increases the immunological risk in potential recipients, whereas a high degree of HLA mismatch is another independent risk factor for poor graft survival (3). In addition, most anti-humoral immunity regimens are associated with severe adverse effects like myelosuppression, hemocytopenia and infection, as well as a significant economic burden. Therefore, a prophylactic anti-humoral immunity strategy is urgently needed for the recipients with high immunologic risks.

B cells mediate humoral immune reaction by producing antibodies, and promote cell-mediated immune responses by acting as antigen-presenting cells. They circulate between secondary lymph tissue and priming organs and facilitate inflammation and immune reaction by secreting cytokines. Current B cell-targeting therapies are focused on either depletion of B cell population (e.g., rituximab) or inhibiting antibody production (e.g., bortezomib). Several ongoing preclinical and clinical trials were investigating the outcome of B cell inhibition in high immunologic risk populations (4, 5), and their preliminary results were marginally good.

Iguratimod (IGU) is a novel disease modification anti-rheumatoid drug (DMARD) with potent anti-inflammatory effects in animal models of arthritis and clinical rheumatoid diseases (6). It suppresses antibody production by directly inhibiting the NF-κB pathway in B cells (7, 8). Studies have highlighted its protective effects on lupus nephropathy in a mouse model and a small clinical study (9, 10), and a recent randomized clinical trial also showed its efficacy against primary Sjögren’s syndrome (11). In a previous study, we found that IGU mitigated antibody-mediated rejection (ABMR) in a pre-sensitized mouse transplant model (unpublished), which is not surprising given the similarities between autoimmune diseases and graft rejection. Furthermore, IGU exhibited fewer adverse effects in rheumatoid arthritis patients compared to conventional immunomodulators, which indicates its potential as an adjuvant in renal transplantation (12).

There is no clinical report so far on the combination of IGU with classic anti-rejection regimens in human renal transplant patients. Here we conducted a small pilot study to investigate the possibility of adding IGU in highly mismatched renal transplant recipients as adjuvant therapy. The aim of this preliminary study was to evaluate the possible effect and safety of IGU in order to justify a formal clinical trial in the future.

## Methods

### Ethical Statement

The study was approved by the ethics committee of the Affiliated Hospital of Nanjing Medical University (2016-SR-029) and has been registered at www.clinicaltrials.gov (NCT02839941). Written informed consent was obtained from all transplant recipients and recorded in the case report form files. All procedures were performed in accordance with the institutional and national guidelines, and the 1964 Helsinki declaration and its later amendments or comparable ethical standards. The donors were lineal or collateral relatives not beyond the third degree of kinship, or unrelated donors after cardiac death.

### Study Design and Population

This pilot study was a randomized, open-label clinical trial, and all participants were recruited from the Kidney Transplantation Center of the Affiliated Hospital of Nanjing Medical University (Nanjing, China). The inclusion criteria for the patients were as follows: (1) aged 18 to 65 years old, (2) underwent kidney transplantation surgery at least 2 weeks before the enrollment, (3) HLA mismatch with the corresponding donors ≥ 4, (4) stable graft function with serum creatinine level < 1.5 times the upper limit of normal and not more than ±10% after three consecutive follow-up, (5) preoperative PRA < 10%, (6) immunosuppressant level before the enrollment is within the target concentration range, and (7) voluntary participation. Patients with delayed graft function (DGF), primary non-function (PNF), on-going acute rejection, pre-formed DSA, multi-organ transplantation, second kidney transplantation, or any major organ dysfunctions, as well as pregnant or breastfeeding women were excluded.

### Sample Size Calculation and Randomization

Due to the pilot study in nature, no formal sample size estimation was performed. Subjects were enrolled consecutively and assigned a random 3-digit number generated by SPSS (IBM, New York, USA). Patients with an even number were assigned to the IGU group, and those with odd numbers to the control group. The patients or clinicians were not blinded to the grouping. The follow-up physicians observed and recorded all events, and the transplant surgeon made the final decision based on the findings. A pharmacist allocated the drug according to research protocol. Two nephropathologists were masked to groups when reviewing graft biopsy slides.

### Treatment

Patients that received at least one dose of IGU were defined as the modified intention-to-treat (mITT) population. The per-protocol population (PP) included patients that were treated for at least 3 months and followed up, and the protocol biopsy population (PB) had received at least 2 protocol biopsies. Patients in the IGU group received 25 mg oral IGU twice daily [BID] along with the conventional triple immunosuppressive protocol for 52 weeks, and the control group was only administered the routine treatment without IGU. The initial immunosuppression protocol for each patient was prednisone, Tacrolimus (0.05 to 0.1 mg/kg/day, twice daily [BID] reaching target trough level of 10 ng/mL), and mycophenolate mofetil (MMF, 0.75 to 1.0 g twice daily [BID]). Methylprednisolone was intravenously administered at the dose of 500 mg/day on the day of surgery and until 2 days after the transplantation. The dosage was reduced thereafter to 400 mg, 300 mg, 200 mg and then 80 mg over each subsequent day. This was followed by oral administration of 30 mg/day prednisone as maintenance therapy. Furthermore, 20 mg basiliximab (Simulect^®^, Novartis, Switzerland) was intravenously administered 30 min before the surgery and on the fourth day post-transplantation. Once the renal allograft function stabilized, Tacrolimus (target trough level of 6 to 10 ng/mL), MMF (0.5 to 1.0 g twice daily [BID]) and prednisone (25 mg daily initially and then tapering to 5 mg daily within 3 months) were administered as maintenance therapy. The patients that were intolerant to Tacrolimus at the target dose were given low-dose Sirolimus additionally. For acute rejection (AR), 200 mg/day methylprednisolone was intravenously administered for 3 to 5 days as soon as the renal allograft biopsy was performed. All patients received 450 mg/day Ganciclovir for 3 months as preventative therapy.

All patients were requested to receive protocol allograft biopsy on the day of randomization, and 24 and 52 weeks later. Any inexplicable deterioration in allograft function was considered as a sign of clinical rejection and confirmed by the indicated biopsy and pathological examination according to the Banff 2017 criteria (13). The subclinical rejection was defined as histologically AR without concurrent functional deterioration of renal allograft, which was usually detected by protocol biopsy (14). The medical records of the enrolled patients were critically and independently reviewed by two physicians. The weight, height, serum biochemical indices (kidney and liver function markers), blood cell analysis, immunoglobulin levels, DSA and immunological subsets data of each patient were collected prior to enrollment. General consultation, physical examination, blood cell analysis, serum biochemistry analysis, and immunoglobulin quantification were routinely performed every 4 weeks during the follow-up. DSA levels and lymphocyte subsets were analyzed every 3 months. Other tests were performed as necessary. Two independent statisticians analyzed all data. Two physicians recorded all adverse events (AE), along with any laboratory abnormalities with or without clinical symptoms. Severe adverse events (SAE) were reviewed by the senior supervisor to determine whether to remove any patient.

### DSA Assessment

Serum levels of HLA I and II antibodies were detected using the LABScreen TM Single Antigen HLA Class I, Class II and MICA kits (One Lambda, Thermo Fisher, USA) according to the manufacturer’s instructions (15). The signals were read using the Luminex-200 instrument (LABScan 200 FlowAnalyzer, USA) and analyzed with the HLA Fusion TM Version 4.4 (One Lambda, USA). MFI > 500 was considered positive, and MFI > 1000 was deemed clinically significant. Based on the levels of donor HLA-A, -B, -C, -DRB1 and -DQB1 antigens, the corresponding DSA class I and II were determined.

### Outcome

The first primary endpoint was biopsy-proven acute rejection (BPAR) rate, including clinical and subclinical rejection. BPAR rate was evaluated in both mITT and PB populations. The second primary endpoint was functional allograft survival, wherein serum creatine level and estimated glomerular filtration rate (eGFR) were calculated. The Chinses-MDRD equation was used: eGFR=170 × SCr^-0.999^ × age^-0.176^ × serum urea nitrogen^-0.170^ × serum albumin^0.318^ × (0.762 if female) (16). The secondary endpoints were the safety profile, DSA and other indicators. The biosafety was analyzed in the mITT population by reviewing the medical records and categorized according to the National Cancer Institute (NCI) Common Terminology Criteria for Adverse Events (CTCAE) version 4.0 (17). Other indices were evaluated in PP population, and included the incidence of infection, liver function and gastrointestinal symptoms according to the adverse event profile of IGU in the rheumatic population (12). Other secondary endpoints were DSA, T and B cell counts and subtypes, and the serum levels of immunoglobulins and complement factors (C3 and C4).

### Statistical Analysis

The continuous data were presented as means ± standard deviation (SD) and compared using Student *t*-test or non-parametric test for two groups, or analysis of variance (ANOVA) for multiple groups. The categorical data were presented as percentages and compared using chi-squared test or Fisher’s exact method as appropriate. The 1-year rejection-free survival rates were compared using Log-rank test in the Kaplan-Meier method. All statistical analyses were conducted using the Stata 15.0 software (StataCorp, Texas, USA).

## Results

### Patient Selection and Baseline Characteristics

**Figure 1:**
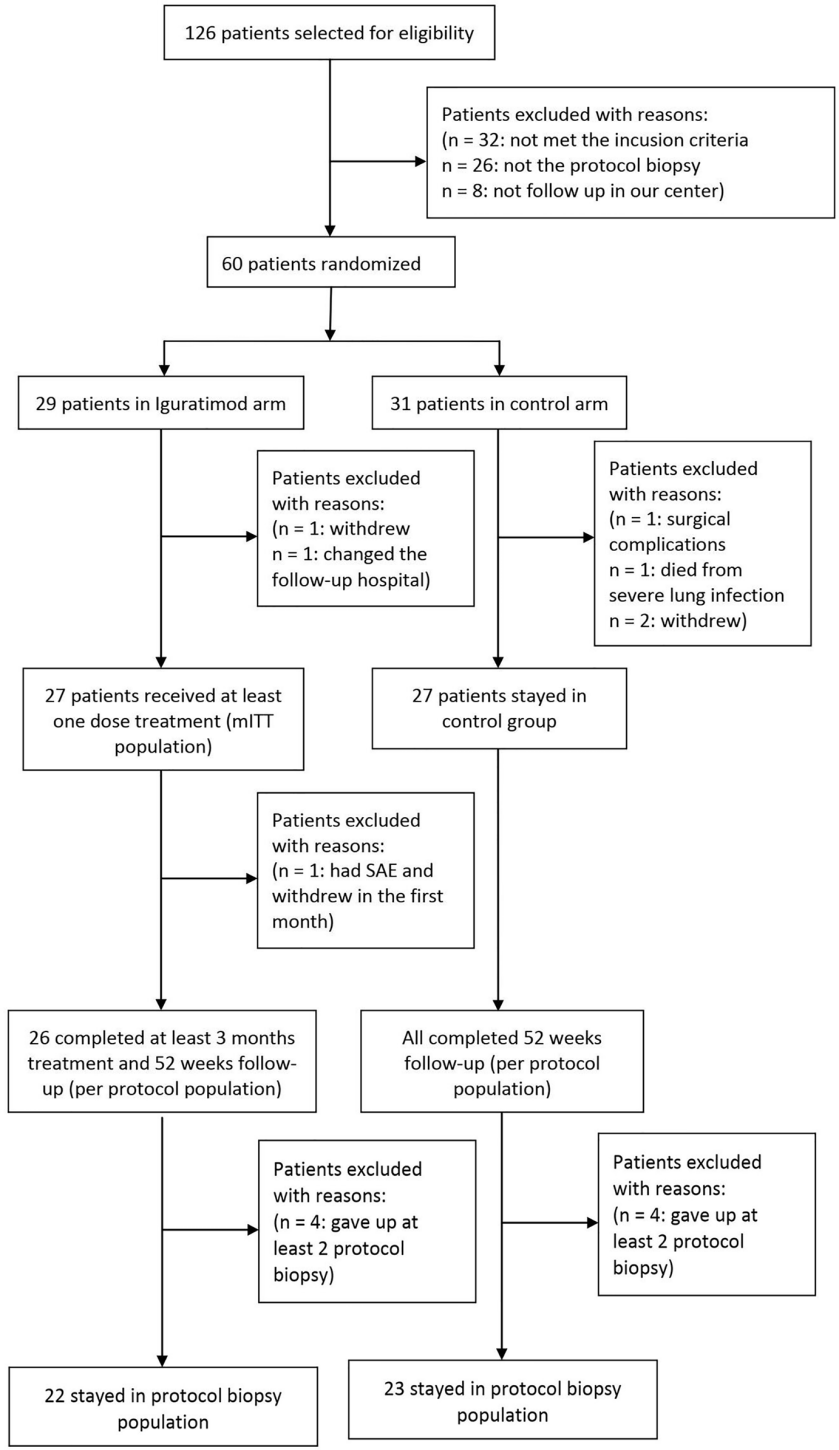
Study profile.

We screened 126 patients who received kidney transplant at our center from Feb 2018 to Oct 2019 and selected 60 patients based on the inclusion criteria. The patients were randomized into the IGU versus non-IGU groups at 1:1 ratio. Three patients (1 in the IGU and 2 in the control group) withdrew voluntarily, one patient in the IGU group was excluded due to residential relocation, one patient in the control group withdrew on account of a severe surgical complication, and one patient in the control group died from pulmonary infection. Finally, 27 patients who received at least one dose of IGU were considered the mITT population in the study arm, and 27 patients were present in the control arm. During the follow-up period, one patient in IGU group withdrew in the first week due to intolerable gastrointestinal AE, which left 26 patients in the PP population of IGU group. Eight patients refused twice for protocol biopsy (4 in each group), which reduced the PB population for IGU and control groups to 22 and 23 patients respectively (**Figure 1**). As shown in **Table 1**, the baseline characteristics of the mITT population was similar in both arms.

**Table 1:** Demographic characteristics of mITT population.

Factors	Level	Control	Iguratimod	*P*-value	Test
**N**		27	27		
**Sex**	Female	7 (26%)	7 (26%)	1.00	Pearson’s chi-squared
	Male	20 (74%)	20 (74%)		
**Age, mean (SD)**		38.9 (9.4)	40.4 (10.0)	0.57	Two sample t test
**Body Mass Index, mean (SD)**		21.7 (2.2)	20.8 (2.8)	0.23	Two sample t test
**Primary Kidney Diseases**	CGN	22 (81%)	22 (81%)	1.00	Fisher’s exact
	IgAN	4 (15%)	5 (19%)		
	Others	1 (4%)	0 (0%)		
**Renal Replacement**	HD	22 (81%)	21 (78%)	0.73	Fisher’s exact
	PD	4 (15%)	6 (22%)		
	Pre-emptive	1 (4%)	0 (0%)		
**Time of RRT, mean (SD)**		38.2 (38.5)	35.0 (34.2)	0.75	Two sample t test
**Mismatched Allele, median (IQR)**		8.0 (6.0, 9.0)	8.0 (7.0, 9.0)	0.89	Wilcoxon rank-sum
**Preformed PRA**	Negative	20 (74%)	15 (56%)	0.15	Pearson’s chi-squared
	Positive	7 (26%)	12 (44%)		
**Transfusion History**	No	25 (93%)	23 (81%)	0.22	Pearson’s chi-squared
	Yes	2 (7%)	5 (19%)		
**Pregnancy History**	0	23 (85%)	22 (85%)	1.00	Fisher’s exact
	1	2 (7%)	3 (11%)		
	2	1 (4%)	1 (4%)		
	3	1 (4%)	0 (0%)		
**Sirolimus**	Not Take	19 (70%)	22 (81%)	0.34	Pearson’s chi-squared
	Take	8 (30%)	5 (19%)		
**Tacrolimus trough**	At day 1	10.3 (2.6)	9.7 (3.0)	0.47	Two sample t test
**ng/mL, mean (SD)**	At week 52	5.1 (1.4)	4.9 (.6)	0.62	Two sample t test
**MMF dose**	At day 1	1.9 (0.2)	1.9 (0.2)	0.38	Two sample t test
**g/day, mean (SD)**	At week 52	1.2 (0.2)	1.2 (0.2)	0.22	Two sample t test

CGN, chronic glomerular nephritis; IgAN, IgA nephropathy; HD, hemodialysis; PD, peritoneal dialysis; RRT, renal replacement therapy; IQR, inter-quartile range; PRA, panel reactive antibody; MMF, mycophenolate mofetil.

### IGU Attenuates Acute Rejection When Administered Along With the Conventional Immunosuppressive Regimen

**Figure 2:**
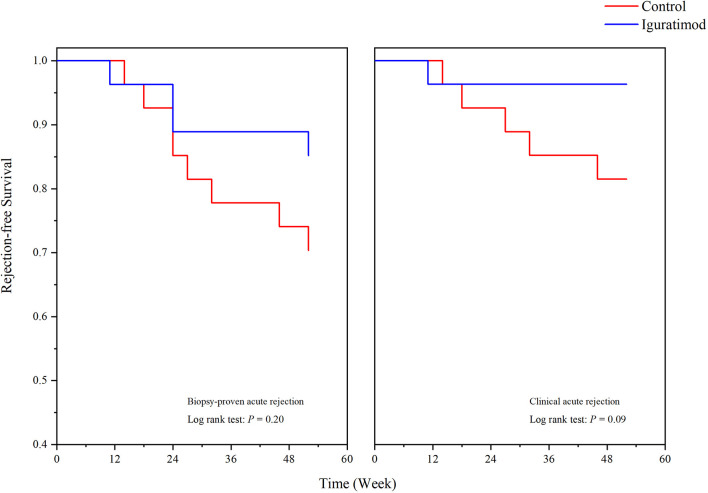
One-year rejection-free survival in Iguratimod and control groups. Biopsy proved acute rejection (BPAR, left) and clinical acute rejection (right) were shown respectively. No statistical significance was detected (*P* = 0.20 for BPAR, and *P* = 0.09 for clinical rejection).

We analyzed the BPAR rate in both the mITT and PB population and the results are shown in **Table 2**. In the IGU group, the overall one-year BPAR rate was 14.8% (4/27), which was lower than the 29.6% (8/27) observed in the control group, but the difference did not reach statistical significance (Pearson’s chi-squared *P* = 0.19). In addition, the IGU arm showed reduction in the incidence of clinical rejection (1 in IGU group *vs*. 5 in the control group, Pearson’s chi-squared *P* = 0.08). In terms of the type of rejection, no pure TCMR occurred in IGU group and three in the control group (Fisher’s exact probability, *P* = 0.24), two ABMR was recorded in each group (*P* = 1.00), and two mixed rejections were recorded in IGU group while three in the control group (*P* = 0.64). Similar results were obtained for the PB populations, which might reflect the true incidence of rejection in one year (34.8% *vs*. 18.2%, *P* = 0.21). Survival analysis further showed that the overall one-year rejection-free survival rate was slightly higher in the IGU arm compared to the control arm (Log-rank *P* = 0.20 for BPAR and *P* = 0.09 for clinical acute rejection; **Figure 2**). In addition, a detailed Banff score was shown in **Table 3**. Taken together, IGU might reduce the first year BPAR in highly mismatched renal transplant patients.

**Table 2:** Univariable analysis of rejection rate between two groups.

Factor	mITT Population			Protocol Biopsy Population		
	Control	Iguratimod	*P*-value	Control	Iguratimod	*P*-value
**N**	27	27		23	22	
**BPAR**	8 (29.6%)	4 (14.8%)	0.19	8 (34.8%)	4 (18.2%)	0.21
**CR**	5 (18.5%)	1 (3.7%)	0.08	5 (21.7%)	1 (4.6%)	0.09
**SCR**	3 (11.1%)	3 (11.1%)	1.00	3 (13.0%)	3 (13.6%)	0.95
**TCMR**	3 (11.1%)	0 (0.0%)	0.24*	3 (13.0%)	0 (0.0%)	0.23*
**ABMR**	2 (7.4%)	2 (7.4%)	1.00	2 (8.7%)	2 (9.1%)	0.96
**Mixed**	3 (11.1%)	2 (7.4%)	0.64	3 (13.0%)	2 (9.1%)	0.67

BPAR, biopsy proved acute rejection; CR, clinical rejection; SCR, subclinical rejection; TCMR, T-cell-mediated rejection; ABMR, antibody-mediated rejection. *Fisher’s exact probability.

**Table 3:** Comparison of detailed pathological parameter between two groups according to Banff 2017 criteria.

Banff parameter	At the beginning			At week 24			At week 52		
	Control	IGU	P value	Control	IGU	P value	Control	IGU	P value
**i**	0.11 ± 0.32	0.22 ± 0.44	0.49	1.00 ± 1.28	0.50 ± 0.91	0.31	0.21 ± 0.43	0.29 ± 0.47	0.68
**t**	0.11 ± 0.32	0.22 ± 0.44	0.49	0.50 ± 0.53	0.42 ± 0.52	0.71	0.29 ± 0.47	0.29 ± 0.47	1.00
**v**	0.05 ± 0.23	0	0.33	0.20 ± 0.42	0.17 ± 0.39	0.85	0.07 ± 0.27	0.07 ± 0.27	1.00
**g**	0.47 ± 0.61	0.22 ± 0.44	0.23	0.60 ± 0.70	0.50 ± 0.67	0.74	0.79 ± 0.70	0.71 ± 0.47	0.75
**ptc**	0.21 ± 0.42	0.22 ± 0.44	0.95	0.60 ± 0.52	0.33 ± 0.65	0.30	0.79 ± 0.58	0.57 ± 0.65	0.36
**C4d**	0.05 ± 0.23	0	0.33	0	0.08 ± 0.29	0.34	0.07 ± 0.27	0.14 ± 0.36	0.56

### Allograft Function Remains Stable During the Administration of IGU

**Figure 3:**
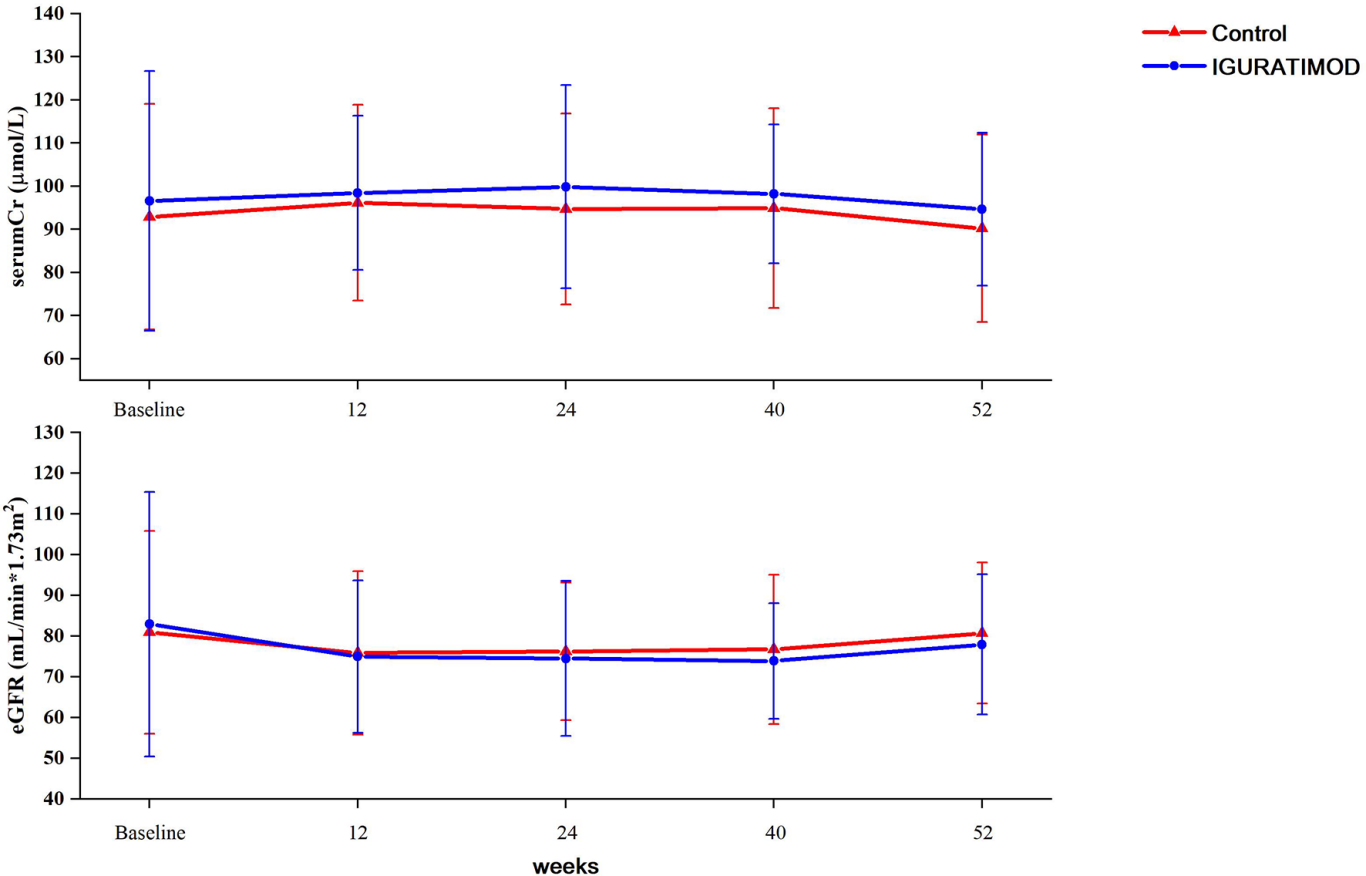
Graft function between two arms. Serum creatinine levels were tested at baseline, 3 months (11~13 week), 6 months (23~25 weeks), 9 months (38~40 weeks) and 12 months (52 weeks). eGFRs were calculated using equation and adjusted to the BSA. Repeated measurement ANOVA method was used to compare the difference between groups. No statistical differences were reached either between two groups (*P* = 0.47 for serum creatinine, and *P* = 0.79 for eGFR).

No graft or patient loss was reported during the 52 weeks of follow-up in both groups, and all patients survived till the end of this trial. The allograft function curve (**Figure 3**) indicated no significant differences between the IGU arm and the control arm (repeated-measure ANOVA, *P* = 0.47 for creatinine and *P* = 0.79 for eGFR). Furthermore, the average allograft function remained stable during the trial in both arms (see **Supplement Table 1**).

### IGU Might Suppresses *De Novo* Generation of DSA

**Figure 4:**
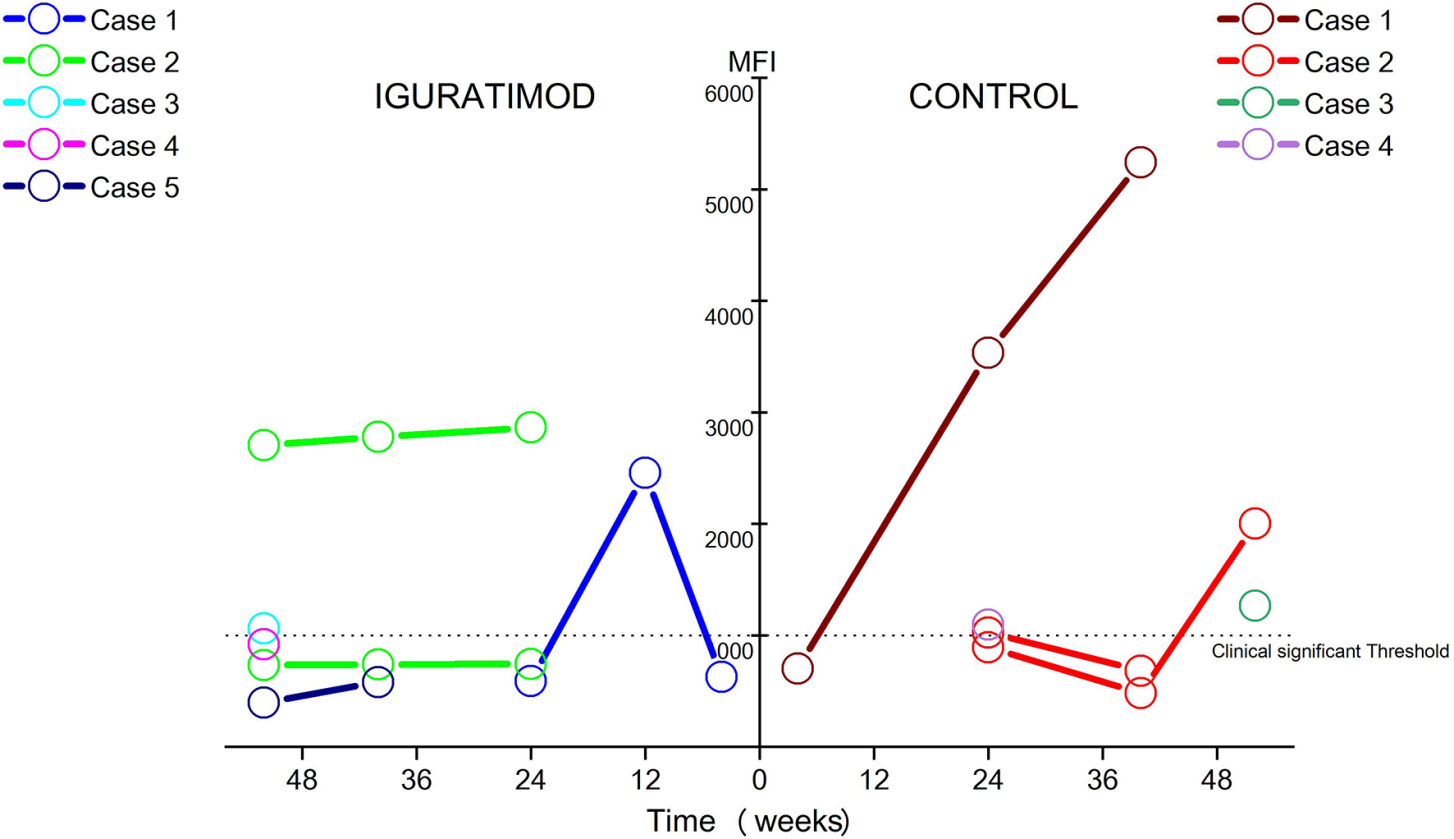
Ebb and flow of *de novo* DSA during follow-up in two groups. Only DSAs with MFI ≥ 500 were detectable DSA and MFI ≥ 1000 were considered clinically significant. In Iguratimod arm, 5 patients developed 6 detectable DSA, only 2 patients (case #2 and case #3) remain clinically significant DSA at last follow-up, with case #3 just borderline. In contrast, 4 patients developed 5 detectable DSA, and 3 of them (case #1, case #2, and case #3) sustained to be clinically significant at the last follow-up, especially with two cases (case #1 and case #2) growing fast in the control group. No statistical difference of DSA incidence was found between two arms (14.8% in the control and 19.2% in the IGU, χ^2^ test: *P* = 0.95). The average MFI for all clinically significant DSA at the last examination was 2048.8 in control group, while 1203.0 in IGU group (*P* = 0.38).

Four cases (14.8%) in the control arm developed five clinically significant *de novo* DSA as opposed to five patients (19.2%) that developed six DSA in the IGU arm, which did not translate to any significant difference (chi-squared test: *P* = 0.95). At the last follow up, however, there were 2 patients with 2 clinically significant DSA in the IGU group, and 3 patients with 3 clinically significant DSA in the control group (**Figure 4**). The average MFI for all clinically significant DSA at the last examination was 2048.8 in the control group compared to only 1203 in the IGU group (*P* = 0.38). These results are indicative of a potential suppressive effect of IGU on the generation of DSA.

### IGU Is Safe for Renal Transplant Recipients

The ALT levels in the control group were 31.6 ± 25.3 IU at baseline, 21.4 ± 16.5 IU at 24 weeks and 18.3 ± 9.7 IU at 52 weeks, compared to 24.7 ± 11.5 IU, 19.9 ± 13.2 IU and 17.2 ± 12.2 IU in the IGU group at the respective time points. No significant difference in ALT level was observed between the two groups at any time point (repeated-measure ANOVA, *P* = 0.20), indicating that IGU does not cause any liver toxicity. Hemoglobin level was elevated after renal transplant as anticipated, but showed a similar trend between both groups (109.5 ± 10.8 g/L, 139.8 ± 16.0 g/L, and 141.0 ± 18.8 g/L at baseline, 24 weeks and 52weeks in the control group *vs*. 104.6 ± 11.4 g/L, 134.9 ± 20.5 g/L, and 138.3 ± 17.0 g/L in IGU group, repeated-measure ANOVA, *P* = 0.27). As shown in **Table 4**, 12 patients in the IGU group reported 18 AEs, and ten patients reported 14 AEs in the control group, which did not amount to a significant difference. Gastrointestinal discomfort was frequent in the IGU group (8 events in IGU group *vs*. 0 in the control group), albeit transient and tolerable. One patient complained of severe gastrointestinal reactions during the first week and discontinued the use of IGU and was therefore excluded from the PP population. No SAE-related death occurred during this trial.

**Table 4:** Patient reported adverse events during the trial.

Adverse effect	mITT population (%)		*P* value
	Control N = 27	IGU N = 27	
**Lung Infection**	7	5	0.40
**Neutropenia**	3	2	1.00
**Headache**	1	1	1.00
**Somnolence**	1	1	1.00
**Insomnia**	1	0	1.00
**Elevated ALT**	1	1	1.00
**Abdominal Distension**	0	1	1.00
**Bloating**	0	3	0.24
**Nausea**	0	3	0.24
**Vomiting**	0	1	1.00
**Total**	14	18	0.40

IGU, Iguratimod; ALT, alanine aminotransferase.

### NK Cells Likely Mediate the Effects of IGU Following Kidney Transplantation

**Figure 5:**
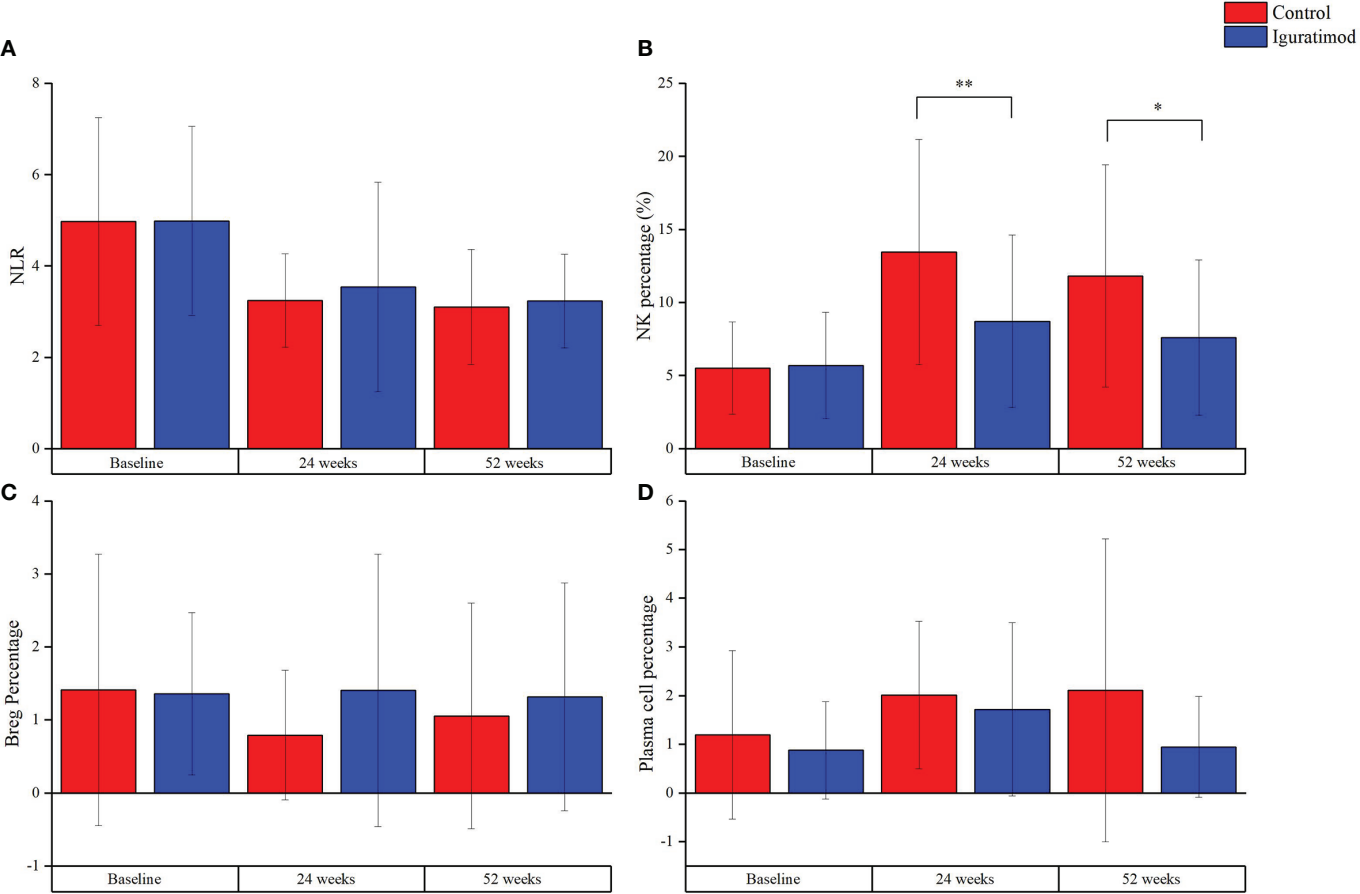
Explorative outcomes at 24 weeks and 52 weeks. **(A)** Neutrophil-to-Lymphocyte ratio, indicating the general inflammation status, decreased with follow-up in both groups. However, there was no statistical significance for the difference between the group (*P* = 0.76). **(B)** The percentage of NK cells, which were CD56^+^CD16^+^ cells measured with flow cytometry, was lower in Iguratimod group than that in the control group at 24 weeks (**P < 0.01) and 52 weeks (*P = 0.01). **(C, D)** represented regulation B (Breg) cells (CD19^+^CD24^+^CD38^+^) and plasma cells (CD19^+^CD24^-^CD38^+^), respectively. There was a trend of higher percentage of Bregs and lower plasma cells in Iguratimod group. But our study was not powerful enough to check it (both *P* > 0.05).

The IGU and control arms did not differ significantly in terms of complement and immunoglobulin levels, as well as T cell counts and subtypes (**Supplement Table 2**). However, the neutrophil-to-lymphocyte ratio (NLR) decreased in time-dependent manner in both groups (*P* = 0.76), which was indicative of a gradual reduction in the general inflammation (**Supplement Table 2** and **Figure 5**). General inflammation status was measured by C-reactive protein (CRP, mg/L). CRP was 6.82 ± 3.78 and 6.82 ± 3.26 in control and IGU group (*P* = 0.998) at baseline. At 24 weeks, CRP was 5.07 ± 1.61 and 5.02 ± 1.15 in control and IGU group respectively (*P* = 0.904). At 52 weeks, CRP was 5.12 ± 1.87 and 4.32 ± 1.86 in control and IGU group respectively (*P* = 0.128). The NK cells were significantly reduced at 24 weeks and 52 weeks in the IGU arm (13.5 ± 7.7% in the control *vs*. 8.7 ± 5.9% in the IGU, *P* < 0.01 at 24 weeks and 11.8 ± 7.6% in the control *vs*. 7.6 ± 5.3% in the IGU, *P* = 0.01 at 52 weeks), which was accompanied by a slight elevation in the regulatory B cells (Breg) and decreased plasma cell counts (both *P* > 0.05). Taken together, IGU likely modulates both innate and humoral immunity in renal transplant patients, and the NK cells may have a crucial role.

## Discussion

This pilot study indicated that the novel DMARD IGU might augment the standard immunosuppressive regimen in renal transplant patients with a high HLA mismatch rate, and decreased the clinical BPAR in one year after transplantation. To our best knowledge, this is the first clinical study to show the safety and efficacy of this drug in this population.

The immunosuppressive effect of IGU in the renal transplant recipients was consistent with its therapeutic activity in RA patients. Along with abating the rejection rate, IGU also affected *de novo* DSAs, which is indicative of its inhibitory effect on the host immune response. However, our pilot study cannot categorically demonstrate a potential modulatory effect of IGU in humoral immunity. Furthermore, our *in vivo* study conducted in rat renal transplant model with antibody-mediated rejection showed significant improvement intervened by IGU (see **Supplementary Material**). The role of humoral immunity in solid organ transplantation has gained considerable attention in recent years. Although depletion of pre-formed DSA can desensitize transplant recipients, little is known regarding the impact of preventing AMR in high-risk recipients. Some immunosuppressive drugs that are effective against autoimmune diseases can prevent allograft rejection as well. For instance, the anti-B cell antibody belimumab mitigates the symptoms of systemic lupus erythematosus and has tested in renal transplant recipients (5). Despite the reduction in the one-year incidence of rejection, IGU treatment did not translate into a functional benefit. This can be partly attributed to sub-clinical rejection, which is characterized by stable graft function but may eventually progress to clinical rejection. Another possibility is that acute rejection is often reversible under proper treatment, and lead to graft function recovery.

Studies show that IGU exerts its immunomodulatory effect by targeting B and T lymphocyte subsets (18, 19). We did not observe any significant changes in the T cell count or subtypes, although IGU treatment led to a slight elevation in Breg cells and a decrease in plasma cells, which indicates a potential effect on B cell differentiation. In our previous study using rat renal transplant model with ABMR and mice secondary skin transplant model, IGU significantly increased the Breg subset during B lymphocyte differentiation. There are reports that IGU can directly or indirectly inhibit immunoglobulin production without affecting the activation or proliferation of B lymphocytes (7, 9, 19). However, we did not see any notable change in IgG levels at this human trial. We hypothesize that the direct effect of IGU on B lymphocytes differentiation into Breg may lead to an impact on Th17 cells, regulatory T cells (Treg), monocytes and invariant natural killer T (iNKT) cells. Interestingly, we did not observe any change in the CD19+ or CD20+ B cell subsets after IGU intervention in human subjects or animal models, which further underscores its role in modulating of B lymphocyte differentiation to Breg subset.

IGU treatment led to a significant decrease in the NK cell population, which has been implicated in both the adaptive and innate immune responses in renal transplantation. Maria et al. showed that NK cells exerted an enhanced killing activity after kidney transplantation in an HLA mismatch-dependent manner (20). Furthermore, Hidalgo et al. found that NK transcripts were enriched in renal allograft biopsies with ABMR and inflammation-related renal injuries (21). We observed an increase in the CD56^+^CD16^+^ NK cell population in the peripheral blood at 24 and 52 weeks post-transplantation compared to baseline (2-4 weeks post-transplantation), which was consistent with the findings of Neudoerfl et al. (22). NK cells recognize self HLA I molecules through the KIR receptors. This inhibitory pathway could contain the killing effect to self-cells. But when they encounter allograft cells with different HLA I molecules, this mechanism breaks. Since our patients were highly HLA mismatched, NK cells might have some roles in modulating rejection responses. IGU intervention led to a significant reduction in the peripheral NK cell counts, which is the first report of a direct association between IGU and NK cell activity. Some studies have demonstrated an interaction of NK cells with Th17 cells in arthritis as well as other inflammatory diseases (23, 24). Therefore, it remains to be elucidated whether NK cells contribute to allograft rejection alone or in combination with other immune cells such as Bregs, and whether IGU can suppress this pathway.

Except for NK cells count, no inflammation markers (such as CRP) were significantly different at follow-up visits in our study. However, there seems to be a trend that lower CRP was achieved in IGU group at 52 weeks. Neutrophil-to-lymphocyte ratio was gradually decreased in both groups along follow-up, which indicated the inflammation process went into remission with time after transplantation. Local inflammation markers rather than systemic markers should be detected in graft biopsy samples to show the significance of inflammation process in allograft injury in further study.

Masako et al. reported that 4.5% of RA patients receiving long-term IGU had elevated serum urea (12). We found that IGU was overall safe in the renal transplant recipients, and the most frequent AEs were transient gastrointestinal reactions. In our mITT population, only one patient dropped from the trial due to SAE. The liver function and allograft function were both stable, which indicates the long-term usage potential of IGU. Some patients did have a slight elevation in liver enzymes, which were normalized upon suitable therapy. Furthermore, the postoperative infection rate was also not affected, indicating that IGU does not impair the host immune response to pathogens. The long-term effect of IGU on the bone density of the recipients should also be investigated. Nevertheless, the safety profile of IGU is more encouraging compared to conventional immunomodulators.

Our study has some limitations that ought to be mentioned. First, our study was a very small sample pilot study without formal sample size calculation. So, there were no strong conclusions can be deduced from our results. Only some trends could be implied in the current study. Second, as an open-label, blank control trial, some intrinsic biases were inevitable. In addition, HLA mismatch was not considered a strong risk factor for *de novo* DSA and ABMR, which undermined the ability to show its superiority in preventing ABMR, as we had postulated before the trial. Furthermore, several patients did not complete the scheduled biopsy, which may have underestimated the real incidence of rejection. However, our results showed a promising strategy that by adding an anti-inflammation drug to conventional immune suppressive regimen, alloimmune reaction might be further contained without significant physiologic and economic burden. These results warrant a formal clinical study of IGU as a potential adjuvant in high risk renal transplant recipients.

In conclusion, IGU might reduce the occurrence of allograft rejection and the onset of *de novo* DSA in highly HLA-mismatched renal transplant recipients. The safety profile was similar to that observed in the RA population. Although the functional benefit of IGU was not obvious within one-year of follow-up, it is a promising immunomodulating drug in a specific renal transplant recipient population. A large-scale, well-designed, randomized controlled study should be performed to identify our conclusions.

## Data Availability Statement

The raw data supporting the conclusions of this article will be made available by the authors, without undue reservation.

## Ethics Statement

The studies involving human participants were reviewed and approved by the ethics committee of the Affiliated Hospital of Nanjing Medical University (2016-SR-029). The patients/participants provided their written informed consent to participate in this study.

## Author Contributions

JT: study design, manuscript preparation. LS: study design and data collection. ZW: data interpretation and manuscript preparation. HC: data collection and data interpretation. ZJH: data collection and data interpretation. HZ: study design. HY: sample collection and data interpretation. ZKH: data interpretation. SF: sample collection. XJ: sample collection and data collection. RT: study design and manuscript preparation. MG: study design, funding, manuscript revision. All authors contributed to the article and approved the submitted version.

## Funding

This work was supported by the National Natural Science Foundation of China [grant numbers 82070769, 81900684, 81870512, 81770751, 81570676], Project of Jiangsu Province for Important Medical Talent [grant number ZDRCA2016025], the “333 High-Level Talents Project” in Jiangsu Province [grant numbers BRA2017532, BRA2016514, BRA2015469], the Standardized Diagnosis and Treatment Research Program of Key Diseases in Jiangsu Province [grant number BE2016791], the Open Project Program of Health Department of Jiangsu Province [grant number JSY-2-2016-099], Jiangsu Province Natural Science Foundation Program [grant number BK20191063].

## Conflict of Interest

The authors declare that the research was conducted in the absence of any commercial or financial relationships that could be construed as a potential conflict of interest.

## Publisher’s Note

All claims expressed in this article are solely those of the authors and do not necessarily represent those of their affiliated organizations, or those of the publisher, the editors and the reviewers. Any product that may be evaluated in this article, or claim that may be made by its manufacturer, is not guaranteed or endorsed by the publisher.

## Abbreviations

TCMR, T cell-mediated rejection; DSA, donor-specific antibody; HLA, human lymphocyte antigen; ABMR, antibody-mediated rejection; IGU, Iguratimod; DMARD, disease modification anti-rheumatoid drug; DGF, delayed graft function; PNF, primary non-function; mITT, modified intention-to-treat; PP, per-protocol population; PB, biopsy population; MMF, mycophenolate mofetil; AR, acute rejection; AE, adverse events; SAE, severe adverse events; BPAR, biopsy-proven acute rejection; GFR, estimated glomerular filtration rate; NCI, National Cancer Institute; CTCEA, Common Terminology Criteria for Adverse Events; SD, standard deviation; ANOVA, analysis of variance; Breg, regulatory B cells; Treg, regulatory T cells; iNKT, invariant natural killer T.
