# Supplementary material for: Efficacy and Safety of Iguratimod Supplement to the Standard Immunosuppressive Regimen in Highly Mismatched Renal Transplant Recipients: A Pilot Study
Source: Front Immunol. 2021 Nov 23;12:738392. doi: 10.3389/fimmu.2021.738392 (PMC8650225; doi:10.3389/fimmu.2021.738392)
Supplement: Supplementary file 1 [file DataSheet_1.docx]

**Supplementary material: Results of IGU on rat renal transplant model with antibody-mediated rejection (AMR)**

1. **Identification of rat renal transplant model with AMR.**

We have established and validated the rat renal transplant model with ABMR. Please refer to published article (**Construction and identification of an antibody-mediated rejection model after kidney transplantation. Hong Chen, Jiajun Zhou, Zijie Wang, et al. J Nanjing Med Univ，2019,39(05):677-681**).

1. **IGU attenuates the progression of AMR in vivo.**

Kidney of donor was transplanted to recipients when DSAs levels reached highest after skin transplantation. As shown in Supplemental Figure 1, kidney of Skin group showed the similar pathological changes as normal rats. Kidneys in the AMR group began developing inflammation in PTC and glomerulitis at first day after kidney transplantation. As transplantation days increasing, PTC inflammation, glomerulitis and interstitial hemorrhage showed serious trend. Compared with the AMR group, the pathologic process of transplanted kidney in the Iguratimod group was greatly decreased. In this research, the dynamic process of pathological change can be clearly observed. C4d and IgG staining on PTC were intense and diffuse in AMR group, however, Iguratimod group was relatively mild and concentrated (Supplemental Figure 2 and Figure 3).
