# Supplementary figures and images for: Efficacy and Safety of Iguratimod Supplement to the Standard Immunosuppressive Regimen in Highly Mismatched Renal Transplant Recipients: A Pilot Study
Source: Front Immunol. 2021 Nov 23;12:738392. doi: 10.3389/fimmu.2021.738392 (PMC8650225; doi:10.3389/fimmu.2021.738392)

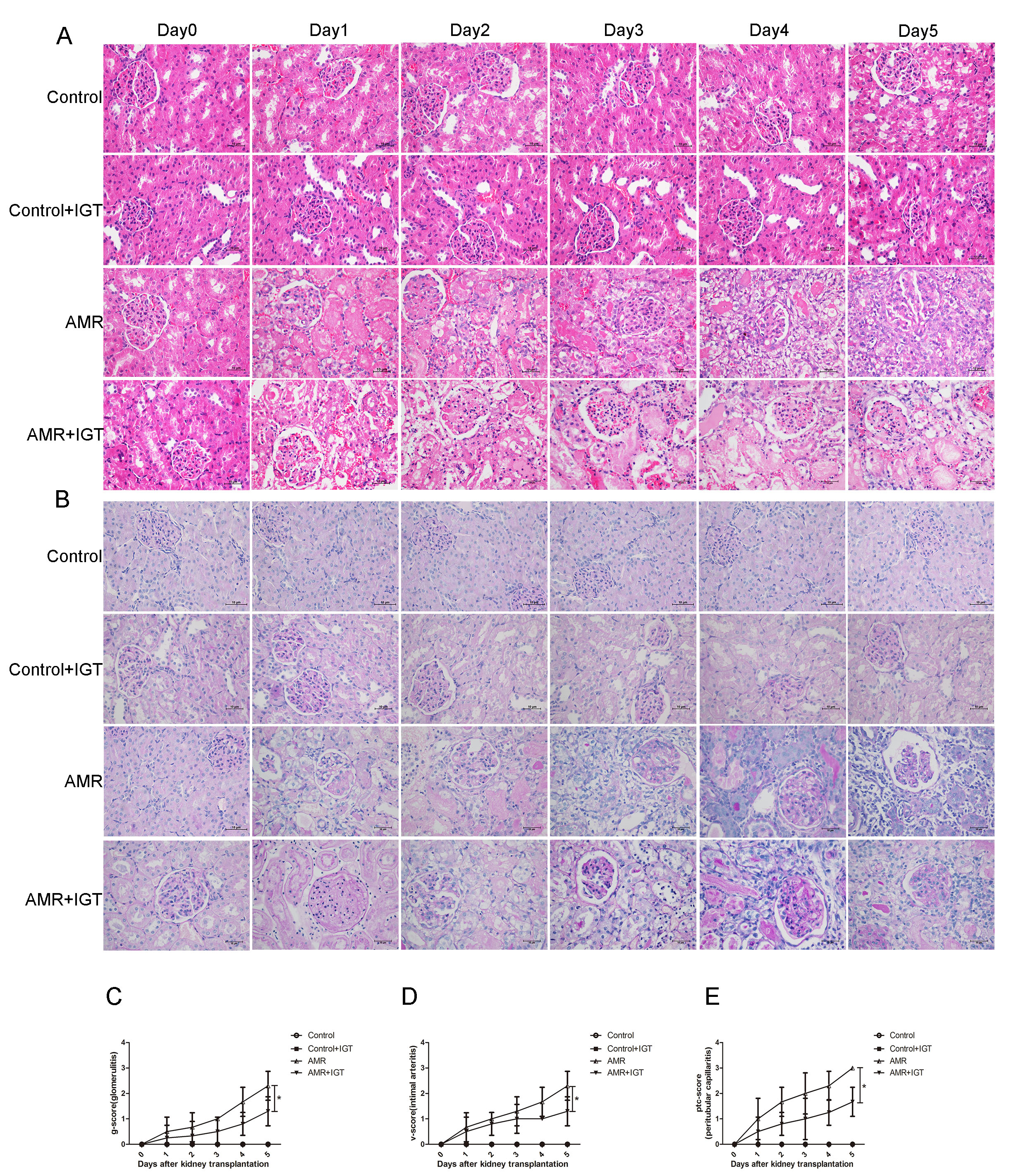

Supplement: Supplementary file 2 [file Image_1.jpeg]

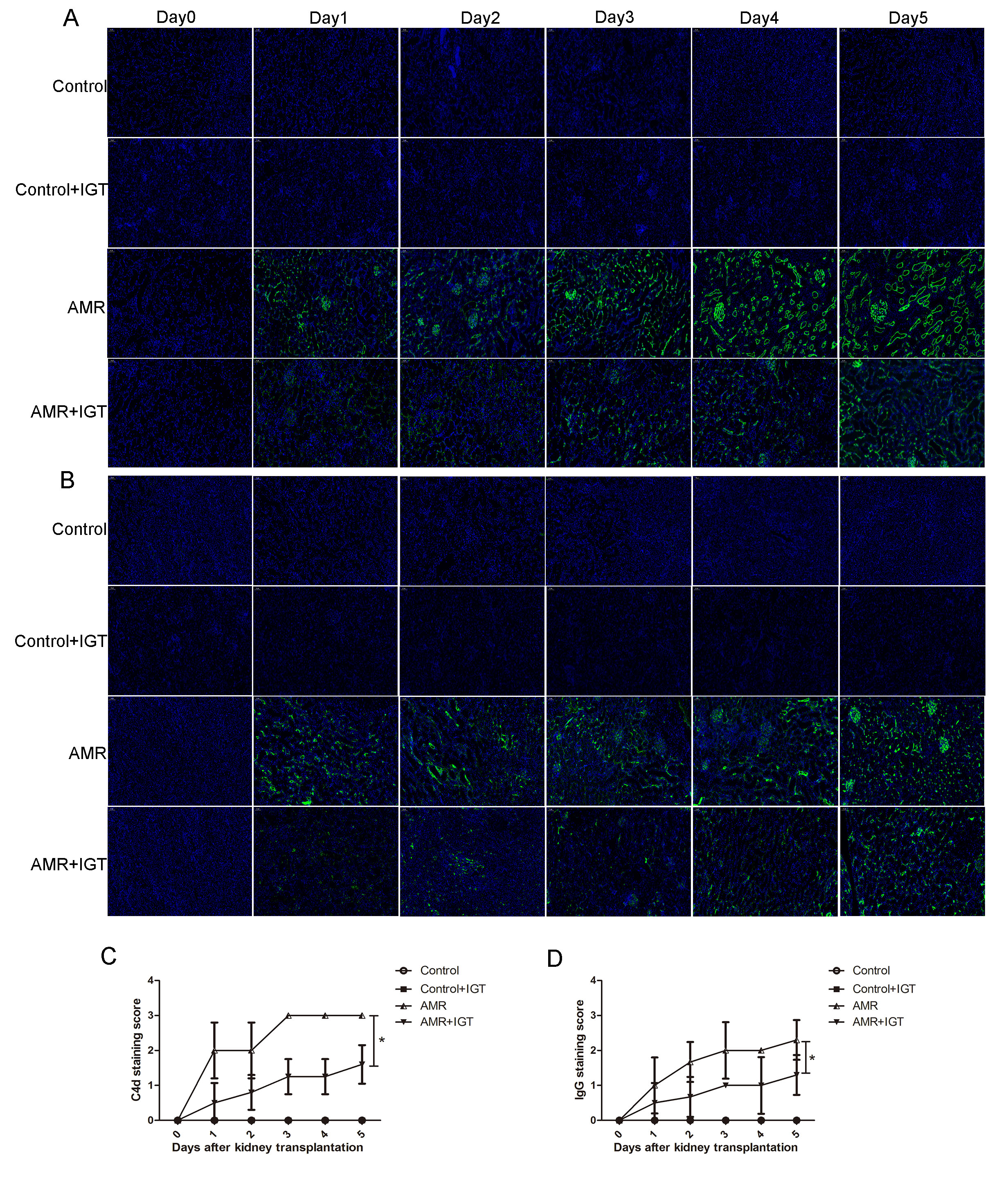

Supplement: Supplementary file 3 [file Image_2.jpeg]

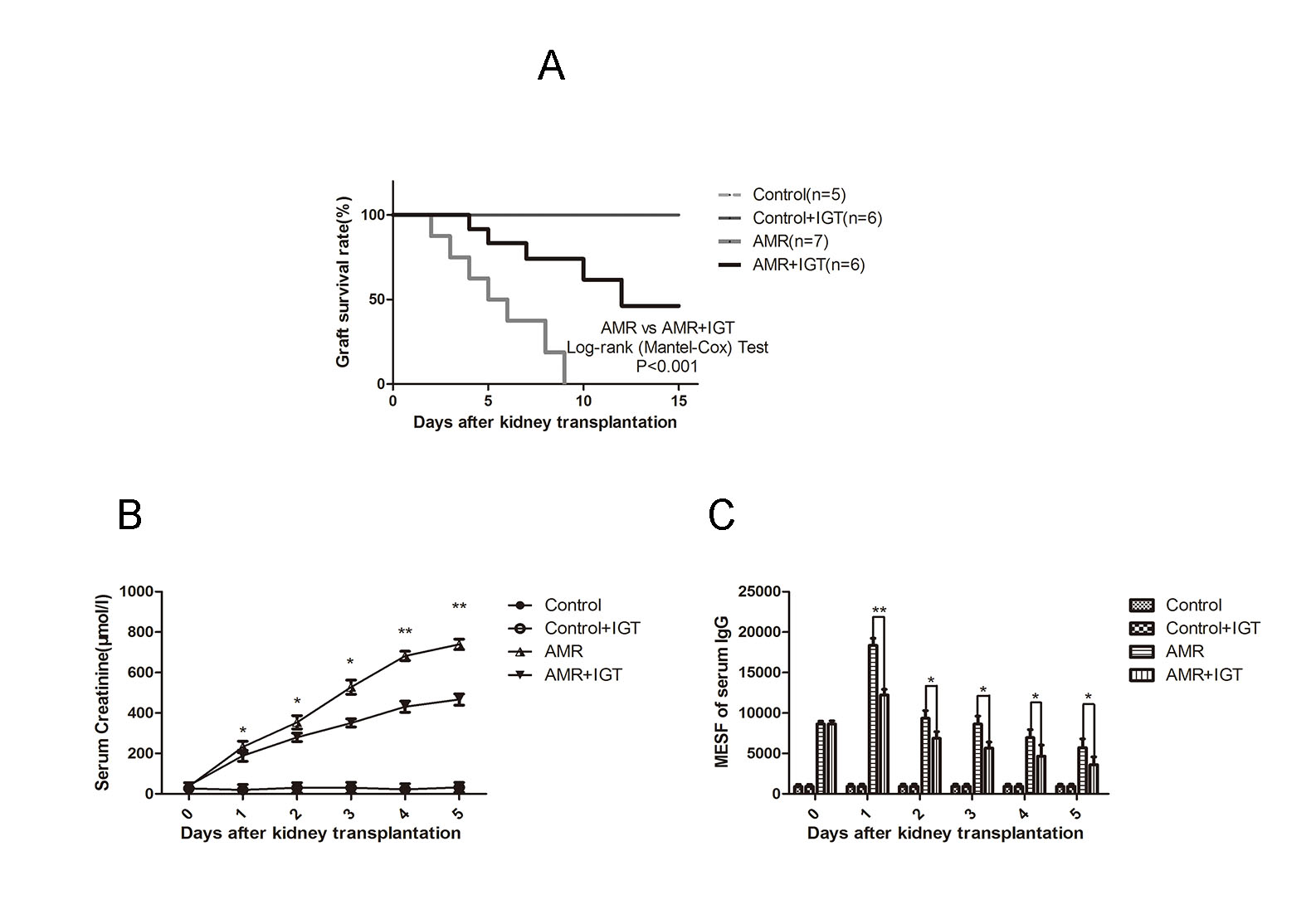

Supplement: Supplementary file 4 [file Image_3.jpeg]
