# Supplementary material for: Efficacy and Safety of Iguratimod Supplement to the Standard Immunosuppressive Regimen in Highly Mismatched Renal Transplant Recipients: A Pilot Study
Source: Front Immunol. 2021 Nov 23;12:738392. doi: 10.3389/fimmu.2021.738392 (PMC8650225; doi:10.3389/fimmu.2021.738392)
Supplement: Supplementary file 5 [file Table_1.docx]

**Supplement Table 1. Serum creatinine level and estimated GFR at each visit**

|  |  | **Baseline** | **12 weeks** | **24 weeks** | **38 weeks** | **52 weeks** |
| --- | --- | --- | --- | --- | --- | --- |
| **SCr** | control | 92.0 ± 26.3 | 96.0 ± 23.1 | 94.9 ± 22.6 | 95.1 ± 23.6 | 90.2 ± 22.2 |
|  | IGU | 97.3 ± 29.8 | 98.4 ± 17.5 | 99.4 ± 23.2 | 97.9 ± 15.8 | 94.5 ± 17.4 |
| **eGFR** | control | 81.4 ± 25.2 | 75.7 ± 20.4 | 75.6 ± 16.9 | 76.2 ± 18.5 | 80.3 ± 17.6 |
|  | IGU | 82.3 ± 32.0 | 75.2 ± 18.4 | 75.2 ± 19.0 | 74.4 ± 14.2 | 78.4 ± 17.1 |

SCr was presented in μmol/L; eGFR was calculated by Modification of Diet in Renal Disease (MDRD) equation, adjusted for body surface area, and presented in ml/min•1.73m^2^.

**Supplement Table 2. Explorative outcomes.**

|  | **Group** | **Baseline** | **24 weeks** | **52 weeks** |
| --- | --- | --- | --- | --- |
| **NLR** | control | 5.0 ± 2.3 | 3.3 ± 1.0 | 3.1 ± 1.3 |
|  | IGU | 5.0 ± 2.1 | 3.5 ± 2.3 | 3.3 ± 1.0 |
| **NK cells ratio (%)** | control | 5.5 ± 3.2 | 13.5 ± 7.7 | 11.8 ± 7.6 |
|  | IGU | 5.7 ± 3.6 | 8.7 ± 5.9 | 7.6 ± 5.3 |
| **Breg cells ratio (%)** | control | 1.4 ± 1.9 | 0.8 ± 0. 9 | 1.1 ± 1.5 |
|  | IGU | 1.4 ± 1.1 | 1.4 ± 1.9 | 1.3 ± 1.6 |
| **Plasma cells ratio (%)** | control | 1.2 ± 1.7 | 2.0 ± 1.5 | 2.1 ± 3.1 |
|  | IGU | 0.9 ± 1.0 | 1.7 ± 1.8 | 1.0 ± 1.0 |
| **CD4+ T cells ratio (%)** | control | 44.8 ± 8.7 | 41.3 ± 9.1 | 42.8 ± 9.8 |
|  | IGU | 46.7 ± 6.8 | 45.5 ± 6.8 | 46.3 ± 7.1 |
| **CD8+ T cells ratio (%)** | control | 27.1 ± 6.9 | 29.0 ± 5.3 | 31.2 ± 9.2 |
|  | IGU | 25.1 ± 6.6 | 30.2 ± 7.1 | 29.6 ± 8.1 |
| **Th17 cells ratio (%)** | control | 0.15 ± 0.03 | 0.14 ± 0.03 | 0.15 ± 0.04 |
|  | IGU | 0.14 ± 0.03 | 0.14 ± 0.04 | 0.14 ± 0.04 |
| **Treg cells ratio (%)** | control | 8.2 ± 1.7 | 8.1 ± 1.9 | 8.1 ± 2.1 |
|  | IGU | 8.3 ± 1.4 | 8.0 ± 1.8 | 7.9 ± 2.0 |
| **B cells ratio (%)** | control | 17.2 ± 9.6 | 8.4 ± 4.3 | 8.6 ± 5.3 |
|  | IGU | 17.3 ± 5.6 | 8.6 ± 3.7 | 8.8 ± 3.2 |
| **IgG level (g/L)** | control | 7.7 ± 1.3 | 8.8 ± 1.5 | 8.8 ± 1.4 |
|  | IGU | 7.6 ± 1.8 | 8.0 ± 2.6 | 8.3 ± 2.2 |
| **Complement C3 level (g/L)** | control | 0.9 ± 0.2 | 1.0 ± 0.1 | 1.0 ± 0.2 |
|  | IGU | 0.9 ± 0.1 | 0.9 ± 0.1 | 0.9 ± 0.2 |
| **Complement C4 level (g/L)** | control | 0.2 ± 0.1 | 0.2 ± 0.1 | 0.2 ± 0.1 |
|  | IGU | 0.2 ± 0.1 | 0.2 ± 0.1 | 0.2 ± 0.1 |

NLR: neutrophil-to-lymphocyte ratio; Breg: regulation B cell; Treg: regulation T cell
